# The prognostic impact of prevailing definitions of periprocedural myocardial infarction in patients undergoing coronary artery bypass grafting

**DOI:** 10.1093/ehjqcco/qcaf043

**Published:** 2025-06-20

**Authors:** Brian Swinnen, Michal J Kawczynski, Alma M A Mingels, Joachim E Wildberger, Casper Mihl, Martijn W Smulders, Jos G Maessen, Can Gollmann-Tepeköylü, Samuel Heuts

**Affiliations:** Department of Cardiothoracic Surgery, Maastricht University Medical Centre (MUMC+), P. Debyelaan 25, Maastricht 6229HX, the Netherlands; Department of Radiology and Nuclear Medicine, Maastricht University Medical Centre (MUMC+), Maastricht, the Netherlands; Cardiovascular Research Institute Maastricht (CARIM), Maastricht University, Maastricht, the Netherlands; Department of Cardiothoracic Surgery, Maastricht University Medical Centre (MUMC+), P. Debyelaan 25, Maastricht 6229HX, the Netherlands; Cardiovascular Research Institute Maastricht (CARIM), Maastricht University, Maastricht, the Netherlands; Cardiovascular Research Institute Maastricht (CARIM), Maastricht University, Maastricht, the Netherlands; Central Diagnostic Laboratory, Maastricht University Medical Centre (MUMC+), Maastricht, the Netherlands; Department of Radiology and Nuclear Medicine, Maastricht University Medical Centre (MUMC+), Maastricht, the Netherlands; Cardiovascular Research Institute Maastricht (CARIM), Maastricht University, Maastricht, the Netherlands; Department of Radiology and Nuclear Medicine, Maastricht University Medical Centre (MUMC+), Maastricht, the Netherlands; Cardiovascular Research Institute Maastricht (CARIM), Maastricht University, Maastricht, the Netherlands; Department of Radiology and Nuclear Medicine, Maastricht University Medical Centre (MUMC+), Maastricht, the Netherlands; Cardiovascular Research Institute Maastricht (CARIM), Maastricht University, Maastricht, the Netherlands; Department of Cardiology, Maastricht University Medical Centre (MUMC+), Maastricht, the Netherlands; Department of Cardiothoracic Surgery, Maastricht University Medical Centre (MUMC+), P. Debyelaan 25, Maastricht 6229HX, the Netherlands; Cardiovascular Research Institute Maastricht (CARIM), Maastricht University, Maastricht, the Netherlands; Department of Cardiac Surgery, Innsbruck Medical University, Innsbruck, Austria; Department of Cardiothoracic Surgery, Maastricht University Medical Centre (MUMC+), P. Debyelaan 25, Maastricht 6229HX, the Netherlands; Cardiovascular Research Institute Maastricht (CARIM), Maastricht University, Maastricht, the Netherlands

**Keywords:** Coronary artery bypass grafting, Myocardial infarction, Periprocedural myocardial infarction, Meta-analysis, Frequentist, Bayesian

## Abstract

**Aims:**

Several contradictory definitions have been proposed for the diagnosis of periprocedural myocardial infarction (PMI) after coronary artery bypass grafting (CABG). The aim of this study was to assess the prevalence of PMI and to identify the definition of PMI with the most relevant prognostic impact.

**Methods and results:**

In this systematic review and meta-analysis, the search was conducted in thee electronic databases (MEDLINE & PubMed Central, Cochrane Library, Embase). The primary definitions of interest comprised the universal definition of myocardial infarction (UDMI; UDMI-3/4) and Society for Cardiovascular Angiography and Interventions (SCAI) definition. The primary outcomes were the prevalence of PMI and its prognostic impact, expressed in hazard ratios (HRs) and 95% confidence intervals (CIs). The frequentist framework was employed for the primary analysis, and a secondary analysis was performed under a Bayesian framework. Ten studies were included (*n* = 21 203 patients). The prevalence of PMI was 17.5% (95%CI 9.5–29.8%) according to SCAI, and 3.2% (95%CI 1.6–6.2%) according to UDMI-3/4. The pooled HR of the SCAI definition for freedom from all-cause mortality was 1.60 (95%CI 1.18–2.16) and the HR was 2.54 (1.62–4.00) for UDMI-3/4 (*P*-for-interaction = 0.097). The posterior probability of exceeding an HR of 1 was >99% for both definitions, while the probability of the UDMI-3/4 exceeding the mean HR of SCAI was 96.4%. The results were robust across sensitivity analyses.

**Conclusion:**

The prevalence of PMI is markedly higher when diagnosed according to SCAI criteria in CABG patients. The UDMI criteria define PMI with the most relevant prognostic impact in CABG patients.

## Graphical Abstract

**Graphical Abstract:**
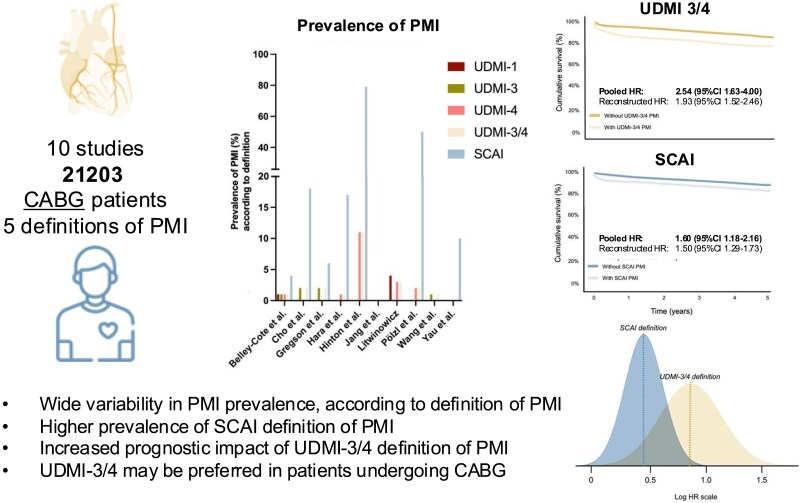

Key Learning Points What is already known?

- Contemporary and prevailing definitions of periprocedural myocardial infarction (PMI) following coronary artery bypass grafting (CABG) differ markedly.
- The Universal Definition of Myocardial Infarction (UDMI) and the definition as proposed by the Society of Cardiovascular Angiography and Interventions (SCAI) even propose contradictory criteria to diagnose PMI.
- It remains uncertain which definition of PMI is prognostically most relevant.

What this study adds

- There is considerable variability in the prevalence of PMI after CABG, which is notably higher when diagnosed according to SCAI criteria.
- All definitions are independently associated with prognosis, but the UDMI defines PMI with the most relevant prognostic impact.

## Introduction

Myocardial injury as expressed by release of cardiac biomarkers occurs through different mechanisms in patients undergoing coronary artery bypass grafting (CABG), albeit with arguable prognostic relevance.^1^ Moreover, the definition of periprocedural myocardial infarction (PMI) as a complication of CABG is heavily debated.^2^ Thus far, several definitions have been proposed by different societies and task forces. The prevailing definitions comprise the universal definition of myocardial infarction (UDMI)^3^ and its sequentially updated versions (UDMI-3^4^ and UDMI-4,^5^) the definition as stated by the Society for Cardiovascular Angiography and Interventions (SCAI),^6^ and the definition suggested by the second Academic Research Consortium (ARC-2)^7^ (*Table 1*). However, these definitions are contradictory on several critical aspects, namely; their preference for different biomarkers [MB isoenzyme of creatine kinase (CK-MB) or cardiac troponin (cTn), whether I or T], varying biomarker concentrations thresholds that are indicative of PMI, and the relevance of isolated biomarker concentration elevations.^1,2,5,6^ It is also uncertain whether the same criteria can be applied to patients undergoing CABG and percutaneous coronary interventions (PCI). Recently, Paolucci and colleagues demonstrated that the prevalence of PMI was the lowest using the SCAI definition in PCI patients, while the risk of death during follow-up was highest in patients with a SCAI-PMI.^8^ It remains uncertain whether these findings are extrapolatable to CABG patients.

**Table 1:** Overview of historical and contemporary definitions of periprocedural myocardial infarction

Definition	Cardiac biomarker	Cut-off values of cardiac biomarkers	Ancillary criteria
UDMI (within 72 h)^3^	cTn	If normal baseline: >5×URL	At **least** one of the following criteria: New pathological Q waves or new LBBB Graft occlusion or new native coronary occlusion, confirmed through cardiac angiography New loss of viable myocardium or new regional wall motion abnormality
	or CK-MB		
UDMI-3 (within 48 h)^4^	cTn	If normal baseline: >10 × URL	At **least** one of the following criteria: New pathological Q waves or new LBBB Graft occlusion or new native coronary occlusion, confirmed through cardiac angiography New loss of viable myocardium or new regional wall motion abnormality
UDMI-4 (within 48 h)^5^	cTn	If normal baseline cTn: >10×URL	At **least** one of the following criteria: New pathological Q wave^a^ Graft occlusion or new native coronary occlusion, confirmed through cardiac angiography New loss of viable myocardium or new regional wall motion abnormality
		If elevated preprocedure cTn that are stable (≤ 20% variation) or falling: increase of >20%, up to 48 h following surgery, always accompanied by a > 10xURL elevation	
SCAI (within 48 h)^6^	CK-MB, cTn if CK-MB is not available	**Isolated** biomarker increase: CK-MB: > 10 ULN. cTn: > 70 × ULN.	New Q waves in ≥2 contiguous leads or new persistent LBBB
		**In presence** of ECG-abnormalities:	
		CK-MB: > 5 ULN.cTn: > 35 × ULN.	
ARC-2^7^	cTn	Absolute increase of cTn >35 × URL	At least one of the following criteria: New pathological Q-waves Flow-limiting angiographic complications New loss of viable myocardium or new regional wall motion abnormality

ARC, academic research consortium; SCAI, society of cardiovascular angiography and interventions; UDMI, universal definition of myocardial infarction.

^a^ST-segment elevation with reciprocal ST-segment depression or other specific ECG patterns may also be reliable findings of a potential ischaemic event.

Therefore, the current study aims to perform a systematic review and meta-analysis of the literature to evaluate the prevalence and prognostic impact of the various PMI definitions in patients undergoing CABG.

## Methods and materials

### Design and protocol

The current study was designed as a systematic review and meta-analysis, and preregistered in the PROSPERO database (CRD42024550121, dated 2 June 2024).^9^ Deviations from the original protocol were reported in Supplementary material online, *S1*. We adhered to the 2020 PRISMA statement (see Supplementary material online, *S2*).^10^

### Definitions of periprocedural myocardial infarction

*Table 1* provides an overview of historical and prevailing definitions of PMI, including the first UDMI,^3^ UDMI-3,^4^ UDMI-4,^5^ the SCAI definition,^6^ and ARC-2.^7^ Of note, as UDMI-3 and UDMI-4 are virtually identical, they are being analysed both separately and in a combined fashion in the current study, as proposed previously.^8^ In case both UDMI-3 and UDMI-4 are reported by an included study, UDMI-4 is preferred over UDMI-3.

### Eligibility criteria

Studies published between conception of the queried databases and 5 May 2024 were eligible for inclusion. Inclusion criteria comprised: (i) studies describing cohorts of patients undergoing (isolated) CABG, and (ii) reporting one or more of the prevailing definitions of (P)MI (UDMI,^3^ UDMI-3,^4^ UDMI-4,^5^ SCAI,^6^ ARC-2^7^), and (iii) reporting on mortality/survival (at any timepoint) while establishing a prognostic relation between the prevalence PMI and these outcomes [through relative measures such as the odds ratio, relative risk, or hazard ratio (HR)]. Of note, both (sub-studies of) randomized controlled trials (RCTs) and observational and retrospective studies were eligible, though case reports, reviews, and other meta-analyses were excluded. In addition, we included both single- and two-armed studies, conditional on the adherence to the other inclusion criteria. To ensure generalizability and homogeneity, studies were excluded when applying any other definition of PMI, or when they only adhered to parts of the prevailing definitions of PMI (for example, only biomarker thresholds but not ancillary criteria). Furthermore, cohorts containing patients undergoing other cardiac surgical procedures and/or patients undergoing percutaneous interventions were excluded.

### Information sources and search strategy

Three electronic databases (MEDLINE & PubMed Central, Cochrane Library, Embase) were systematically queried from conception until 5 May 2024. The search was constructed by two experienced authors (M.J.K. and S.H.).

The systematic and reproducible search strategy is presented in Supplementary material online, *S3*, and contains disease-, treatment-, and outcome-associated terms such as ‘PMI’, ‘CABG’, and ‘survival’ (and all possible alternative spellings).

### Selection process and data collection

Two authors performed the selection process independently (B.S. and S.H.), using the semi-automated web-based application Rayyan (http://rayyan.qcri.org).^11^ The initial phase concerned the systematic screening of eligible articles based on title and abstract, after which the second stage comprised an in-depth evaluation of full-texts. Any disagreement was eventually resolved through consultation of a third independent author (M.J.K.).

Two authors performed the data collection process in a predefined Excel worksheet (B.S. and S.H.; Supplementary material online, *S4*).

### Outcomes and effect measures

The primary outcomes of the current study were (i) the prevalence of PMI according to the various definitions of PMI, and (ii) their association with freedom from all-cause mortality. These prevalences were expressed in pooled percentages with corresponding 95% confidence intervals (CIs). The association with freedom from all-cause mortality was expressed in pooled (adjusted) HRs with corresponding 95% CIs, or credible intervals (CrIs, when Bayesian statistical models were applied). In case both unadjusted and adjusted HRs were presented by studies, adjusted HRs were preferred.

### Risk of bias assessment

As both single- and two-armed studies were eligible for inclusion, one universal quality appraisal tool was applied, which is modifiable based on the number of study arms; the Newcastle-Ottawa Scale (NOS, Supplementary material online, *S1*). As described previously,^12,13^ the NOS can be adapted if only one study arm is presented, by removing one of four questions in the selection process (i.e. Question 2 in *Selection—selection of the nonexposed cohort*). This assessment was performed by two authors independently (B.S. and S.H.).

### Data synthesis

Continuous variables that were not presented in means and standard deviations (SDs) by the original articles were converted using Wan’s method.^14^ The prevalences of PMI were pooled per definition separately (and in conjunction for UDMI-3/4) using a random-effects model (inverse variance weighting). Statistical heterogeneity among studies was objectively assessed and quantified using the *I*^2^-metric and *τ*^2^. In the second step, the reported HRs were pooled using frequentist methods (random-effects, DerSimonian and Laird estimator). These pooled analyses were performed for all PMI definitions. In addition, as the Bayesian statistical framework allows the estimation of posterior probabilities of various effect size thresholds, these HRs were pooled using hierarchical Bayesian models as well (Bayesian random-effects model). Of note, these pooled analyses were *only* performed for the UDMI-3/4 and SCAI definitions (as the SCAI and 3rd/4th version of the UDMI are contemporary definitions, ARC-2 was unfortunately infrequently reported, and the first UDMI is outdated). Assuming normal distributions, the log HR was used as a convenient effect measure for these analyses, and the log HR were re-converted to the HR to facilitate intuitive interpretation. These analyses were performed under a minimally informative prior *N*[0,2], implying no difference between groups with a wide distribution, and a prior for heterogeneity (*τ*) with a default inverse-gamma distribution (*α* = 1, *β* = 0.15).

The prognostic relevance of UDMI-3/4 and SCAI were primarily compared. This comparison was performed again using a frequentist model (based on the effect estimate comparison method by Altman and Bland in interaction *P*-values^15^), and by the use of the Bayesian statistical framework. For the Bayesian analysis, we estimated the posterior probabilities of the SCAI definition exceeding the prognostic relevance of UDMI-3/4 at predetermined thresholds (i.e. an HR of >1.0, > the mean HR of UDMI-3/4, and > the lower and upper limit of the 95%CrI of UDMI-3/4), and vice versa. In addition, Bayes Factors were calculated to quantify the strength of the evidence (1, no evidence; 1–3, anecdotal evidence; 3–10, moderate evidence; 10–30, strong evidence; > 30, very strong evidence).^16^

As a sensitivity analysis, we analysed individual patient data reconstructed from Kaplan–Meier (K–M) curves (based on the method by Liu *et al*.^17^) and presented these in pooled K–M curves (with a comparison between patients with and without PMI, using a frequentist univariable Cox-regression model).

All analyses were performed using the R statistical environment (R statistics, version 4.2.2; R Foundation for Statistical Computing), using the ‘meta’ package, and in JASP (JASP, version 0.19.0, Mac), for the Bayesian analyses.

### Publication bias assessment

A publication bias assessment was conducted for the prognostic value of the UDMI-3/4 and SCAI definitions. This evaluation was based on visual inspection of the funnel plots, and statistically tested by Egger’s regression (for which a *P*-value <0.05 denoted presence of statistically significant publication bias).

## Results

### Study selection and characteristics

The systematic search yielded 3616 hits (573 duplicated removed). We screened 3043 articles based on title/abstract, after which 66 studies were evaluated in-depth based on full texts. Eventually, 10 studies were included for final analysis (reasons for exclusion are presented in the PRISMA 2020 flowchart in Supplementary material online, *S5*).

The 10 studies^18–27^ were published between 2008 and 2022. Four studies were posthoc analyses of RCTs [namely; the CORONARY trial,^18^ the EXCEL trial,^20^ the SYNTAX(ES) trial,^21^ and the PREVENT IV trial,^26^] while six studies had a retrospective/prospective observational character.^19,22–25,27^ The first UDMI was reported by three studies,^18,19,23^ UDMI-3 by four studies,^18–20,27^ UDMI-4 by five studies,^18,21,22,24,25^ a combination of either UDMI-3 or UDMI-4 (UDMI-3/4) by eight studies,^18–22,24–26^ and the SCAI definition by seven studies^18–22,25,26^ (*Table 2*). Of note, the study by Yau *et al*.^26^ (posthoc analysis PREVENT-IV) was published in 2008 (before the release of the SCAI definition) but applied criteria that were in complete agreement with the SCAI definition.^6^ As such, Yau *et al*. was included for the analysis of the SCAI definition. Finally, follow-up was reported within a range of 1.0–10.7 years (pooled mean follow-up 3.8 years, SD 3.0 years).

**Table 2:** Study characteristics

Study and year	Type of study	Number of patients enrolled (*n*)	Number CABG patients (*n*)	Definitions of PMI used	Mean follow up (years)	Biomarkers used
Belley-Cote, 2019^18^	Posthoc analysis of RCT (CORONARY Trial)	4752	4752	UDMI-1, UDMI-3, UDMI-4, SCAI	1	Different cTn assays and CK-MB
Cho, 2017^19^	Observational (registry)	7697	3183	UDMI-1, UDMI-3, SCAI	4.8 ± 4.2	CK-MB
Gregson, 2020^20^	Posthoc analysis of RCT (EXCEL Trial)	1858	923	UDMI-3, SCAI	5	cTn (49.5% availability) and CK-MB
Hara, 2020^21^	Post hoc analysis of RCT (SYNTAX Trial)	1652	795	UDMI-4, SCAI	10.7 ± 2.5	CK-MB
Hinton, 2022^22^	Prospective, observational	488	138	UDMI-4, SCAI	1	hs-cTnI and CK-MB
Jang, 2016^23^	Retrospective, observational	927	367	UDMI-1	3.7 ± 2.8	CK-MB
Litwinowicz, 2022^24^	Retrospective, observational	4642	4642	UDMI-4	5.1 ± 2.1	cTn and CK-MB
Pölzl, 2022^25^	Retrospective, observational	2829	2829	UDMI-4, SCAI, ARC	5	hs-cTnT and CK-MB
Wang, 2013^27^	Retrospective, observational	560	560	UDMI	1.8 ± 0.6	hs-cTnT
Yau, 2008^26^	Posthoc analysis of RCT (PREVENT IV TRIAL)	3014	3014	SCAI	2	CK-MB

CI, confidence intervals; SCAI, society of cardiovascular angiography and interventions; UDMI, universal definition of myocardial infarction.

Of note, only one study reported the outcomes of the ARC-2 definition.^25^ Therefore, this definition was not eligible for a pooled analysis.

In total, the 10 studies comprised 21 203 patients undergoing CABG (data on UDMI: *n* = 8302, UDMI-3: *n* = 9418, UDMI-4: *n* = 12 899, UDMI-3/4: *n* = 17 565, SCAI: *n* = 15 377). Supplementary material online, *S6*–*S8* present the patient and procedural characteristics in detail.

### Study quality assessment

Supplementary material online, *S9* presents the risk of bias assessment, according to the NOS. The final risk of bias evaluation ranged from *intermediate* (two studies^22,26^) to a *low risk* of bias (eight studies^18–21,23–25,27^). All studies were submitted to final analysis of their outcomes.

### Pooled prevalence of periprocedural myocardial infarction

**Figure 1:**
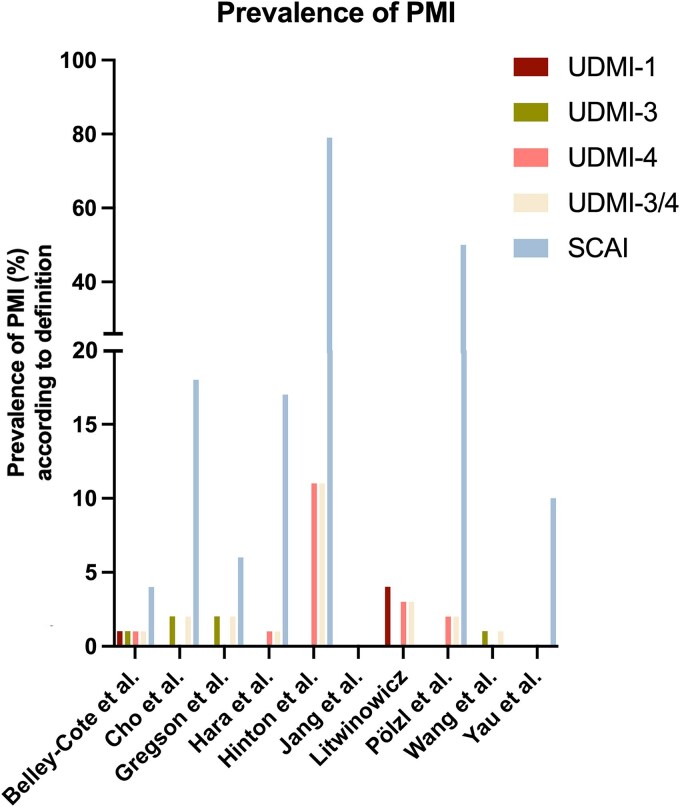
Prevalence of PMI according to the various definitions in the different studies. UDMI-3 and UDMI-4 were combined as these definitions are nearly identical. If a study reported on both UDMI-3 and UDMI-4, the prevalence of UDMI-4 was preferred over UDMI-3. SCAI, society of cardiovascular angiography and interventions; UDMI, universal definition of myocardial infarction.

The prevalence of PMI per definition per study is presented in *Figure 1*. The pooled prevalences of all definitions are presented in *Table 3*. Notably, the prevalence of UDMI-3/4 was 3.2% (95%CI 1.6–6.2%) and of the SCAI definition 17.5% (95%CI 9.5–29.8%). All definitions exhibited the presence of statistically significant heterogeneity (*I*^2^ all above 93%, *P* < 0.001). Subgroup analyses for separate biomarkers could not be performed.

**Table 3:** Pooled prevalence of PMI according to the various definitions

Definition	Number of studies	Prevalence (%)	95%CI (%)	*I* ^2^ (%)	τ^2^
UDMI-1	3	1.3	0.6–3.1	93.2	0.55
UDMI-3	4	2.4	0.6–9.9	98.9	2.25
UDMI-4	5	2.8	1.3–6.0	97.2	0.79
UDMI-3/4^a^	8	3.2	1.6–6.2	97.9	0.99
SCAI	7	17.5	9.5–29.8	99.5	0.88

^a^UDMI-3 and UDMI-4 were combined as these definitions are nearly identical. If a study reported on both UDMI-3 and UDMI-4, the prevalence of UDMI-4 was preferred over UDMI-3.

CI, confidence intervals; SCAI, society of cardiovascular angiography and interventions; UDMI, universal definition of myocardial infarction.

### Prognostic relevance of the various definitions of periprocedural myocardial infarction

**Figure 2:**
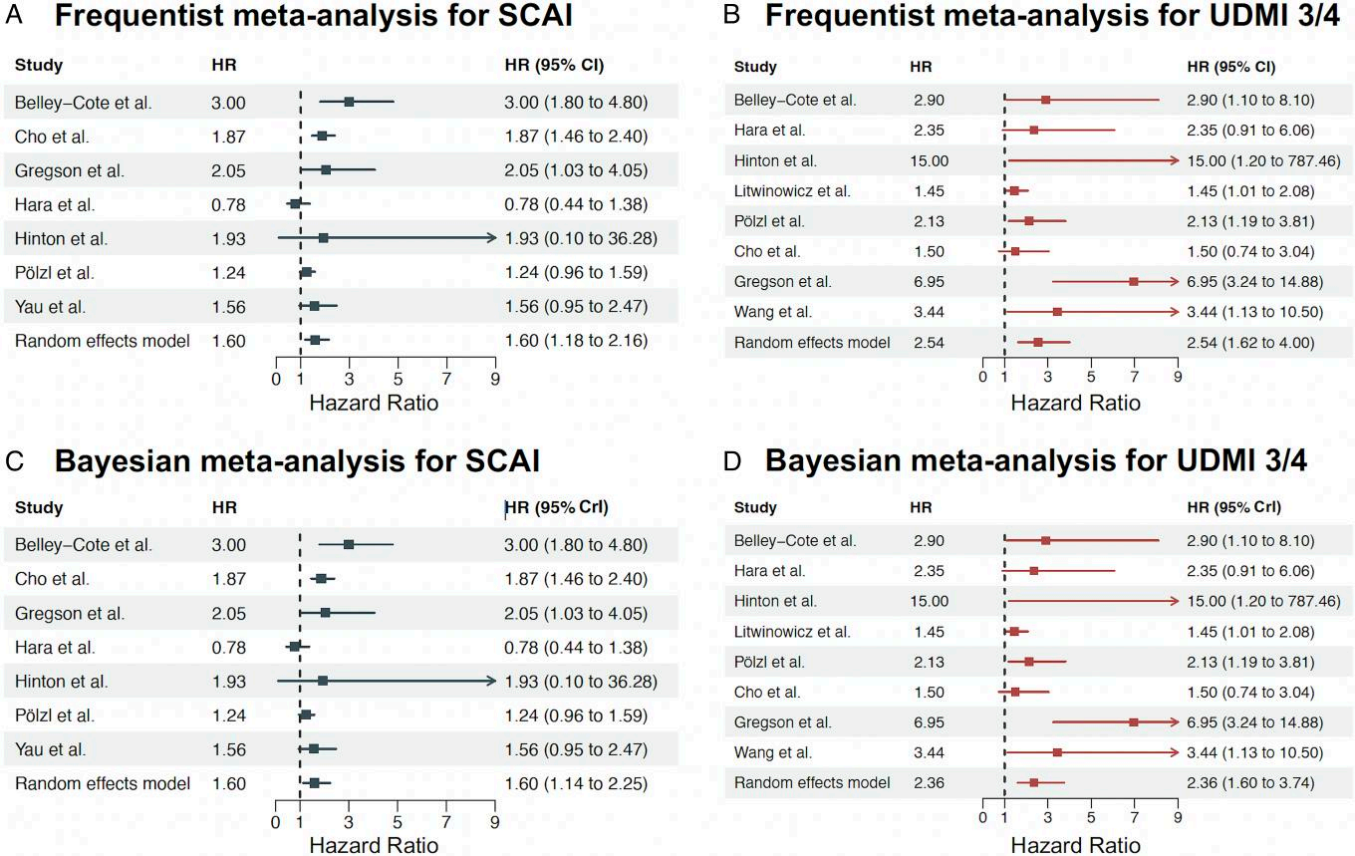
Frequentist and Bayesian meta-analysis of the hazard for mortality during follow-up depending on the use of the SCAI definition (*A*, *C*) and UDMI3/4 definition (*B*, *D*). UDMI-3 and UDMI-4 were combined as these definitions are nearly identical. If a study reported on both UDMI-3 and UDMI-4, the impact of UDMI-4 was preferred over UDMI-3. Bayesian models SCAI: mean log HR; 0.469, SD; 0.170, 95%CrI; 0.134–0.834, Bayes Factor; 3, heterogeneity τ^2^: 0.101. Bayesian models UDMI3/4: mean log HR; 0.858, SD; 0.217, 95%CrI; 0.470–1.325, Bayes Factor; 67, heterogeneity τ^2^: 0.132. CI, confidence interval; CrI, credible interval; HR, hazard ratio; log, natural logarithmic function; SCAI, society of cardiovascular angiography and interventions; UDMI, universal definition of myocardial infarction.

The pooled HR of the SCAI definition for long-term freedom from all-cause mortality was 1.60 (95%CI 1.18–2.16, *I*^2^ = 67%, τ^2^ = 0.094) under a frequentist analysis. In addition, we estimated a mean HR of 1.60 (95%CrI 1.14–2.25, τ^2^ = 0.101) under a Bayesian framework (*Figure 2*, Bayes Factor 3, implying *anecdotal evidence*). For the UDMI-3/4, the combined pooled frequentist HR was 2.54 (1.62–4.00, *I*^2^ = 62%, τ^2^ = 0.228) and we estimated a mean HR of 2.36 (95%CrI 1.60–3.74, τ^2^ = 0.132) in the Bayesian model (*Figure 2*, Bayes Factor 67, implying *very strong evidence*).

The pooled prognostic relevance of UDMI-3 and UDMI-4 separately is reported in Supplementary material online, *S10* [HR 3.36 (95%CI 1.63–6.90) and HR 2.00 (95%CI 1.33–3.01), respectively].

### Universal definition of myocardial infarction-3/4 vs. society of cardiovascular angiography and intervention definition

The interaction *P*-value for the comparison of the SCAI vs. UDMI-3/4 definition was 0.097 (*z* = 1.662), and therefore not statistically significant.

**Figure 3:**
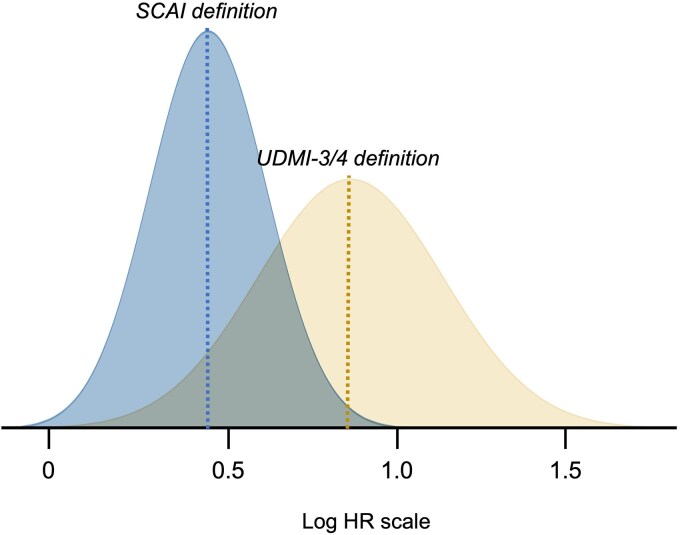
Posterior distribution of the prognostic impact of the UDMI-3/4 and SCAI definitions. HR, hazard ratio; log, natural logarithmic function; SCAI, society of cardiovascular angiography and interventions; UDMI, universal definition of myocardial infarction.

As the Bayesian framework produces a posterior distribution (*Figure 3*), the probability of treatment effect size thresholds can be estimated. The probability of exceeding an HR of 1 was nearly 100% for both definitions (*Table 4*), implying a clinically relevant prognostic impact of both definitions. The posterior probability of the UDMI-3/4 exceeding the mean HR of SCAI was 96.4%, while the probability of SCAI exceeding the mean HR of UDMI-3/4 was only 1.1%. Further probabilities of various thresholds can be appreciated in *Table 4*.

**Table 4:** Posterior probabilities of various studied thresholds on the log HR scale for the UDMI-3/4 and SCAI definition

	UDMI-3/4	SCAI
Mean log HR (Bayesian)	0.86	0.47
95%CrI (log, Bayesian)	0.47–1.33	0.134–0.83
Mean HR (Bayesian)	2.36	1.60
95%CrI (Bayesian)	1.60–3.76	1.13–2.27
*Posterior probabilities*		
> HR 1	100%	99.7%
> mean HR SCAI	96.4%	50.0%
> lower limit 95%CrI HR SCAI	99.9%	97.5%
> upper limit 95%CrI HR SCAI	54.4%	2.5%
> mean HR UDMI-3/4	50.0%	1.1%
> lower limit 95%CrI HR UDMI-3/4	97.5%	49.8%
> upper limit 95%CrI HR UDMI-3/4	2.5%	0%

CI, confidence intervals; CrI, credible interval; HR, hazard ratio; SCAI, society of cardiovascular angiography and interventions; UDMI, universal definition of myocardial infarction.

### Sensitivity analysis and publication bias assessment

**Figure 4:**
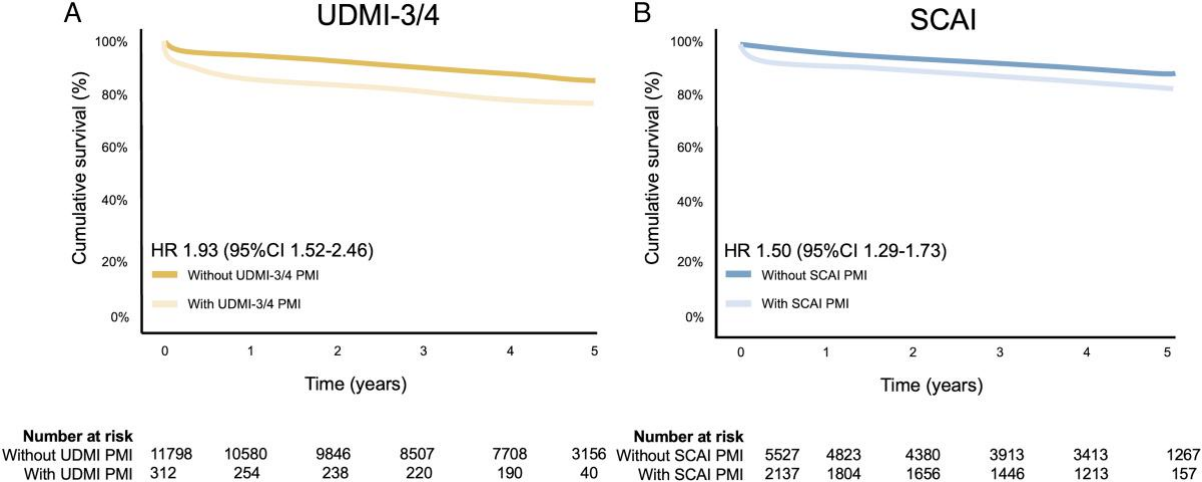
Reconstructed individual patient data comparing patients with and without PMI according to the UDMI-3/4 (*A*) and SCAI definitions (*B*). UDMI-3 and UDMI-4 were combined as these definitions are nearly identical. If a study reported on both UDMI-3 and UDMI-4, the prevalence of UDMI-4 was preferred over UDMI-3. Adequate Kaplan–Meier curves with sufficient data (numbers at risk) were available for five studies for the UDMI-3/4 definitions,^19–21,24,25^ and for four studies for the SCAI definition.^19–21,25^ CI, confidence intervals; HR, hazard ratio; SCAI, society of cardiovascular angiography and interventions; UDMI, universal definition of myocardial infarction.

Adequate K–M curves with sufficient data (including numbers at risk) were available for five studies for the UDMI-3/4 definitions^19–21,24,25^, and for four studies for the SCAI definition.^19–21,25^  *Figure 4* presents the pooled reconstructed survival analyses, which are in line with the overall analyses [UDMI-3/4 HR 1.93 (1.52–2.46), SCAI HR 1.50 (1.29–1.73)].

Publication bias assessment was performed for the two most frequently reported definitions of PMI (UDMI-3/4, SCAI). Supplementary material online, *S11* presents the funnel plots for UDMI-3/4 and SCAI. Egger’s regression test yielded a *P*-value of 0.125 and 0.899, respectively, implying absence of statistically significant publication bias.

## Discussion

The aim of this study was to provide insight into the prevalence and prognostic impact of the prevailing definitions of PMI in patients undergoing CABG in the literature, and to identify the definition of PMI with the most relevant prognostic impact after CABG. The key findings of this study include: (i) a notably higher prevalence of PMI when the SCAI definition was applied, in contrast to a lower prevalence according to the UDMI-3/4 definition, and (ii) a markedly increased prognostic impact of UMDI-3/4 in terms of long-term freedom from all-cause mortality, as compared with the prognostic impact of the SCAI definition of PMI.

The definition of PMI and its impact are among the most widely debated topics in cardiovascular research,^2^ a discussion that was particularly sparked by the publication of the 5-year results of the EXCEL-trial.^28,29^ This debate is not only confined to the academic arena, but is also prevalent in the clinical community, as the application of different definitions leads to a varying PMI rate.^25^ In CABG patients, we observed a PMI-prevalence of 2.4–2.8% according to the UDMI-3 or UDMI-4 definition, which amounts to 17.5% when applying the SCAI definition. Interestingly, if PMI would be defined by the presence of new loss of viable myocardium, as can be assessed by cardiac magnetic resonance imaging (CMR), the prevalence is reported to reach up to 40%, as demonstrated in an elegant study by Selvanayagam and colleagues.^30^ The heterogeneity in these prevalences illustrates the difficulty in determining and diagnosing PMI, while it simultaneously underlines the need for a uniform definition of PMI. Of particular note, such a definition should have a relevant impact on prognosis.

Our results are in sharp contrast with the findings of a recent publication of Paolucci and colleagues, in which the prevalence and prognostic impact of each PMI definition was assessed in patients undergoing PCI.^8^ In their commendable study, the authors calculated a higher pooled prevalence of a UDMI-3/4 PMI (6.9%, 95%CI 5.5–7.2%), as compared with an SCAI-PMI (2.1%, 95%CI 2.3–2.4%) in PCI patients. Instead, in the current analysis, the prevalence of PMI was notably higher when the SCAI definition was applied.^18–22,25^ Since the SCAI definition of PMI does not necessarily require ancillary criteria, such as new onset ECG abnormalities, visual loss of viable myocardium, or proven graft dysfunction, we suggest that the difference in SCAI-PMI prevalence between PCI and CABG may be explained by this criterion. Indeed, during CABG, several mechanisms are in play that can induce (abundant) cardiac biomarker release, which generally originate from noncoronary sources like the use of cardiopulmonary bypass, cardioplegic arrest, and mechanical manipulation.^1^ In addition, these release mechanisms of cardiac biomarkers are generally not associated with (focal) ECG-changes or wall motion abnormalities, and may only induce diffuse myocardial injury.^1^ Consequently, a considerable number of patients may exhibit significantly increased biomarker release after CABG,^12,31^ regardless of the presence of ancillary criteria. All of such patients will be diagnosed with a SCAI-PMI, but not with a PMI according to the UDMI-3/4 criteria.

In their study, Paolucci *et al*. observed a notably increased prevalence of UDMI-PMIs in PCI patients, which may be explained from a pathobiological points of view as well. Noncoronary sources of biomarker release are virtually absent during a PCI procedure, while coronary origins include side-branch occlusions, distal embolization, and stent thrombosis.^32^ Although these complications may be relatively rare, they can intuitively result in focal ECG-changes and echocardiographic abnormalities. Nevertheless, when these complications occur in small or distal vessels, they may only lead to a small increase in CK-MB concentrations (<5xURL) prohibiting the diagnosis of a SCAI-PMI. However, as hs-cTn is generally more abundantly expressed due its increased sensitivity (>10xURL), the threshold to diagnose a UDMI-PMI may be lower. Together, these differences in criteria may explain how the prevalence of SCAI-PMI differs as markedly as observed between PCI and CABG procedures.

Besides variations between definitions and the use of ancillary criteria, the prevalence and impact of PMI can also vary within a definition, when applied to the same population. Inherently, this does not apply to UDMI, as the UDMI only allows cTn assays for a PMI diagnosis. For the SCAI definition, this was recently demonstrated by Piccolo *et al*. in a single-centre analysis of patients undergoing elective PCI.^33^ Although the SCAI definition prefers CK-MB as the biomarker of choice, hs-cTn can also be used when CK-MB is unavailable. Given the increased extent to which hs-cTn may be expressed, different normalized thresholds apply (i.e. > 5xURL with ancillary criteria or >10xURL without, for CK-MB, and >35xURL with supporting evidence or >70xURL without, for hs-cTn). In their study, the authors observed an important prognostic impact of a SCAI-PMI using CK-MB as biomarker (HR 4.27, 95%CI 1.23–14.80), but not with a SCAI-PMI using hs-cTn (HR 2.04, 95%CI 0.94–4.45), in terms of 1-year all-cause mortality. It must be noted that an hs-cTnI assay was used (Abbott ARCHITECT STAT) in that study. As we have recently demonstrated that hs-cTnI is expressed far more abundantly than hs-cTnT (in CABG patients),^31^ it remains unknown whether these findings also apply to SCAI-PMIs diagnosed by use of hs-cTnT. Also, the study by Piccolo *et al*. only included patients undergoing PCI, and it remains questionable whether their results are extrapolatable to CABG patients as well.

As far as we are aware, no study has been published that assessed the pooled prevalence and prognostic impact of prevailing PMI definitions in patients who underwent CABG. Therefore, with this systematic review and meta-analysis, we have attempted to assess which PMI definition is most applicable to CABG patients. According to our results, the diagnosis of a UDMI-3/4-PMI has a more relevant prognostic impact, and may therefore be preferred in CABG patients. Moreover, given the comparable prognostic impact of the UDMI-3/4 in both PCI^8^ and CABG patients [as reflected by the relatively similar (adjusted) HRs as presented in Paolucci *et al*. and the current study], we may even hypothesize that the UDMI-4 could be preferred in study populations that comprise both PCI and CABG patients. Whether our findings justify a refinement or adjustment of these definitions remains to be determined.^2^

We have complemented our conventional (frequentist) analyses with the Bayesian approach, as shown in *Figure 3*. Both definitions had an important prognostic impact, as reflected by the near-100% probability that the distributions exceeded the HR 1.0-cutoff. We further estimated the probability that the mean HR of the UDMI-3/4 definition exceeded the mean HR of SCAI, which was 96%. These complementary analyses support the notion of the improved prognostic impact of the UDMI-3/4, as compared with the SCAI definition, in CABG patients.

It remains to be unravelled why the UDMI-3/4 has such an increased prognostic relevance in CABG patients. We hypothesize that the ancillary criteria such as new regional wall motion abnormalities, ischaemic ECG changes or angiographic graft-failure in UDMI-3/4 are crucial and largely contribute to this impaired prognosis. In this paradigm, the loss of focal viable cardiac tissue through ischaemia and subsequent necrosis could lead to a decrease in cardiac function, heart failure, and arrhythmia during long-term follow-up, which is intuitively related to mortality. This is also in line with previous studies, assessing patients with NSTEMI.^34^ Nevertheless, although both UDMI and SCAI definitions will capture such patients, the SCAI definition’s broader criteria (without ancillary evidence) will also comprise patients without such prognostically relevant features, thereby potentially limiting its overall prognostic impact in CABG patients.

This study uniquely quantifies the prognostic impact of prevailing PMI definitions after CABG, but our findings should be interpreted within the broader context of ongoing challenges in cardiac surgery outcomes research in general, and for PMI in particular. One unresolved issue is the preference for a specific biomarker. For primary MI, (hs-)cTns are superior to other cardiac biomarkers such as CK-MB because of their improved prognostic relevance. Consequently, many centres do not use CK-MB in their practice anymore, limiting the applicability of the SCAI definition. Also, there is an absence of consensus on optimal biomarker thresholds, particularly for high-sensitivity cardiac troponins.^1,2,12,31,35^ Several studies have shown that troponin kinetics post-CABG differ fundamentally from those observed in type 1 MI.^35–38^ Elevations in (hs-)cTns are often driven by extracoronary sources such as myocardial manipulation or cardioplegia-induced injury,^1^ raising concerns about over-diagnosis of PMI when using conventional cut-offs. As such, refined thresholds or dynamic criteria that incorporate biomarker trajectories over time may offer improved specificity.^39^

Furthermore, current definitions of PMI focus primarily on mortality as an endpoint, while important alternative outcomes (such as heart failure and spontaneous MI) are less well reported in registries. This narrow endpoint scope limits our understanding of the true clinical burden of PMI. Also, there is an increasing call for the incorporation of functional imaging endpoints (e.g. CMR-detected infarct size,^30,40^ or myocardial strain abnormalities) as they may better reflect objectively quantifiable myocardial damage.

### Limitations

First, our analysis included different study designs, which could be derived from both posthoc RCT analyses, and retrospective or prospective observational studies. Some of these studies lacked a head-to-head comparison between the PMI definitions. Also, studies with varying follow-up periods were included, ranging between 1 and 10.7 years. This means that the prognostic impact of the PMI definitions was assessed at different timepoints, which may introduce bias. Although the UDMI advocates for the use of cTns, CK-MB was used in many studies instead of cTn.^19–21^ Given this mixed use in biomarkers, it was not feasible to perform subgroup analyses for biomarkers separately. In addition, we performed a study-level meta-analysis and could therefore not analyse actual individual patient information, although we have attempted to mitigate for this limitation by reconstructing these data, if available. Furthermore, we observed considerable (statistical) heterogeneity in the prevalence of PMI within and between studies. Although this can be interpreted as a shortcoming, it also illustrates the academic and clinical problem of heterogeneity in the diagnosis of PMI. Nevertheless, as a consequence, the estimates of the pooled prevalences may be less reliable, although we deem the prognostic analyses valid. Of note, the current study comprises a pooled analysis of observational (and retrospective) data, and there may be a risk of bias because of underreporting of ancillary criteria of ischaemia. Due to the retrospective and—mostly—single-centre nature of the data, central core labs were not used, making results also more prone to bias. Finally, only one study reported the prevalence and impact of the ARC-2 definition,^25^ which could subsequently not be pooled nor assessed. In that study,^25^ the HR for the ARC-2 definition was similar to the HR of the UDMI-4 definition (2.23 and 2.13, respectively), and it has previously been suggested that the ARC-2 definition may be a valuable compromise between UDMI and SCAI.

## Conclusion

By acknowledging the discrepancies such as the prevalence and prognostic impact between the different PMI definitions, this study emphasized the need for a uniform definition of PMI in CABG patients. We observed a considerable variability in the prevalence of PMI, which was notably higher when diagnosed according to SCAI criteria. All definitions were independently associated with impaired prognosis. Based on our findings, albeit within the absence of individual patient data, the criteria as proposed by the 3rd or 4th UDMI define PMI with the highest prognostic impact, and could therefore be preferred in patients undergoing CABG, and in comparative trials.

## Supplementary Material

qcaf043_Supplementary_Data

## Data Availability

The data repository, coding, and all analyses will be made publicly available through https://github.com/samuelheuts/PMI_in_CABG upon publication of this study.
