# Supplementary material for: The prognostic impact of prevailing definitions of periprocedural myocardial infarction in patients undergoing coronary artery bypass grafting
Source: Eur Heart J Qual Care Clin Outcomes. 2025 Jun 20;11(6):847–56. doi: 10.1093/ehjqcco/qcaf043 (PMC12445649; doi:10.1093/ehjqcco/qcaf043)
Supplement: qcaf043_Supplementary_Data [file qcaf043_supplementary_data.docx]

**Supplementary appendix**

Supplement to:

*The prognostic impact of prevailing definitions of periprocedural myocardial infarction in patients undergoing coronary artery bypass grafting*

By:

Brian Swinnen, Michal J. Kawczynski, Alma M.A. Mingels, Joachim E. Wildberger, Casper Mihl, Martijn W. Smulders, Jos G. Maessen, Can Gollmann-Tepeköylü, and Samuel Heuts.

**Content**

Supplementary Material 1 – Reasons for deviation of the PROSPERO protocol 3

Supplementary Material 2 – PRISMA statement checklist 4

Supplementary Material 3 – Detailed search strategy 5

Supplementary Material 4 – Predefined worksheet for data collection 11

Supplementary Material 5 – PRISMA flowchart for study inclusion 12

Supplementary Material 6 – Pre-operative patient characteristics (i) 13

Supplementary Material 7 – Pre-operative patient characteristics (ii) 14

Supplementary Material 8 – Procedural characteristics 15

Supplementary Material 9 – Risk of bias assessment 16

Supplementary Material 10 – Frequentist meta-analysis of UDMI-3 and UDMI-4 17

Supplementary Material 11 – Publication bias assessment 18

References for Supplementary Material 19

**Supplementary Material 1.** Reasons for deviation of the PROSPERO protocol.

| **Deviation of the PROSPERO protocol** | **Reason for deviation** |
| --- | --- |
| No analysis of secondary outcomes such as myocardial infarction, stroke, or repeat revascularization during follow-up. | These outcomes were only rarely reported (i.e., ‘MACCE’ only by 1 study presenting UDMI-4 data, and 3 studies presenting SCAI data). |
| Risk of bias assessment by RoB 2.0 and ROBINS-I tool. | RoB 2.0 did eventually not apply as we only included post-hoc analyses of RCTs and observational studies. Moreover, many studies were single-armed, to which ROBINS-I cannot be adapted. As such, the NOS was elicited, as it can be modified for single-arm studies. |
| Complementary Bayesian analyses. | Primary analyses are still performed under the frequentist framework. However, as the Bayesian framework can produce distributions, the distributions of the HR can be plotted and probabilities of treatment effects can be estimated. Therefore, the Bayesian approach was added to the primary analysis. |
| Subgroup analyses for patients with ‘biomarker criteria only’ and patients undergoing ‘OPCAB’. | Insufficient data. |

*HR: hazard ratio, MACCE: major adverse cardio- and cerebrovascular events, NOS: Newcastle-Ottawa Scale, OPCAB: off-pump coronary artery bypass grafting, RCT: randomized controlled trial, SCAI: society of cardiovascular angiography and interventions, UDMI: universal definition of myocardial infarction.*

**Supplementary Material 2.** PRISMA statement checklist.

**Supplementary Material 3.** Detailed search strategy.

| **Overview** | | |
| --- | --- | --- |
| Database | MEDLINE & PubMed Central | |
| Platform | PubMed | |
| Date of search | 15.05.2024 | |
| Number of results | 2,145 | |
| **Syntax guide** | | |
| [MeSH] | Medical subject headings | |
| **Search** | **Query** | **Items found** |
| **Disease-associated search terms** | | |
| #1 | Type 5 myocardial infarction | 10,453 |
| #2 | Peri-procedural myocardial infarction | 361 |
| #3 | Periprocedural myocardial infarction | 2,081 |
| #4 | Periprocedural myocardial ischemia | 2,433 |
| #5 | Periprocedural ischemia | 1,823 |
| #6 | Procedural myocardial injury | 26,995 |
| #7 | Periprocedural myocardial injury | 459 |
| #8 | #1 OR #2 OR #3 OR #4 OR #5 OR #6 OR #7 | 39,781 |
| **Treatment-associated search terms** | | |
| #9 | Coronary artery bypass grafting | 79,573 |
| #10 | Coronary artery bypass surgery | 79,573 |
| #11 | Coronary artery bypass | 79,573 |
| #12 | Coronary artery bypass[MeSH] | 57,397 |
| #13 | CABG | 22,174 |
| #14 | Coronary artery bypass graft | 79,573 |
| #15 | Coronary bypass surgery | 83,814 |
| #16 | Coronary artery bypass graft surgery | 79,573 |
| #17 | Coronary bypass | 85,178 |
| #18 | Aortocoronary bypass surgery | 76,594 |
| #19 | Aortocoronary bypass grafting | 79,668 |
| #20 | Aortocoronary bypass | 80,194 |
| #21 | Surgical revascularization | 59,019 |
| #22 | Cardio-Thoracic surgery | 5,231 |
| #23 | Cardiothoracic surgery | 73,063 |
| #24 | #9 OR #10 OR #11 OR #12 OR #13 OR #14 OR #15 OR #16 OR #17 OR #18 OR #19 OR #20 OR #21 OR #22 OR #23 | 197,467 |
| **Outcome-associated terms** | | |
| #25 | Mortality | 1,591,198 |
| #26 | Mortality[MeSH] | 427,314 |
| #27 | Survival | 2,692,956 |
| #28 | Survival[MeSH] | 4,941 |
| #29 | All-cause mortality | 69,574 |
| #30 | #25 OR #26 OR #27 OR #28 OR #29 | 2,767,216 |
| **Combined term** | | |
| #31 | #8 AND #24 AND #30 | 2,503 |
| #32 | Filter: year of publications after 2000 | 2,145 |

| **Overview** | | |
| --- | --- | --- |
| Database | Cochrane Library | |
| Platform | Cochrane Library | |
| Date of search | 15.05.2024 | |
| Number of results | 1,340 | |
| **Syntax guide** | | |
| [MeSH] | Medical subject headings | |
| **Search** | **Query** | **Items found** |
| **Disease-associated search terms** | | |
| #1 | Type 5 myocardial infarction | 21,404 |
| #2 | Peri-procedural myocardial infarction | 187 |
| #3 | Periprocedural myocardial infarction | 769 |
| #4 | Periprocedural myocardial ischemia | 243 |
| #5 | Periprocedural ischemia | 367 |
| #6 | Procedural myocardial injury | 278 |
| #7 | Periprocedural myocardial injury | 253 |
| #8 | #1 OR #2 OR #3 OR #4 OR #5 OR #6 OR #7 | 21,962 |
| **Treatment-associated search terms** | | |
| #9 | Coronary artery bypass grafting | 5,965 |
| #10 | Coronary artery bypass surgery | 12,516 |
| #11 | Coronary artery bypass | 14,952 |
| #12 | Coronary artery bypass[MeSH] | 6,802 |
| #13 | CABG | 6,886 |
| #14 | Coronary artery bypass graft | 7,678 |
| #15 | Coronary bypass surgery | 13,142 |
| #16 | Coronary artery bypass graft surgery | 6,492 |
| #17 | Coronary bypass | 15,724 |
| #18 | Aortocoronary bypass surgery | 445 |
| #19 | Aortocoronary bypass grafting | 137 |
| #20 | Aortocoronary bypass | 546 |
| #21 | Surgical revascularization | 1,893 |
| #22 | Cardio-Thoracic surgery | 940 |
| #23 | Cardiothoracic surgery | 4,156 |
| #24 | #9 OR #10 OR #11 OR #12 OR #13 OR #14 OR #15 OR #16 OR #17 OR #18 OR #19 OR #20 OR #21 OR #22 OR #23 | 20,537 |
| **Outcome-associated terms** | | |
| #25 | Mortality | 126,024 |
| #26 | Mortality[MeSH] | 18,869 |
| #27 | Survival | 139,038 |
| #28 | Survival[MeSH] | 183 |
| #29 | All-cause mortality | 16,119 |
| #30 | #25 OR #26 OR #27 OR #28 OR #29 | 224,747 |
| **Combined term** | | |
| #31 | #8 AND #24 AND #30 | 1,844 |
| #32 | Filter: clinical trials | 1,575 |
| #33 | Filter: year of publications after 2000 | 1,340 |

| **Overview** | | |
| --- | --- | --- |
| Database | Embase | |
| Platform | Embase library | |
| Date of search | 15.05.2024 | |
| Number of results | 131 | |
| **Syntax guide** | | |
| [MeSH] | Medical subject headings | |
| **Search** | **Query** | **Items found** |
| **Disease-associated search terms** | | |
| #1 | Type 5 myocardial infarction.af | 18 |
| #2 | Peri-procedural myocardial infarction | 281 |
| #3 | Periprocedural myocardial infarction | 891 |
| #4 | Periprocedural myocardial ischemia | 4 |
| #5 | Periprocedural ischemia | 18 |
| #6 | Procedural myocardial injury | 138 |
| #7 | Periprocedural myocardial injury | 301 |
| #8 | #1 OR #2 OR #3 OR #4 OR #5 OR #6 OR #7 | 1,519 |
| **Treatment-associated search terms** | | |
| #9 | Coronary artery bypass grafting | 36,707 |
| #10 | Coronary artery bypass surgery | 25,464 |
| #11 | Coronary artery bypass | 119,864 |
| #12 | CABG | 40,898 |
| #13 | Coronary artery bypass graft | 96,542 |
| #14 | Coronary bypass surgery | 6,274 |
| #15 | Coronary artery bypass graft surgery | 8,645 |
| #16 | Coronary bypass | 13,586 |
| #17 | Aortocoronary bypass surgery | 898 |
| #18 | Aortocoronary bypass grafting | 384 |
| #19 | Aortocoronary bypass | 2,851 |
| #20 | Surgical revascularization | 4,777 |
| #21 | Cardio-Thoracic surgery | 21,352 |
| #22 | Cardiothoracic surgery | 85,412 |
| #23 | #9 OR #10 OR #11 OR #12 OR #13 OR #14 OR #15 OR #16 OR #17 OR #18 OR #19 OR #20 OR #21 OR #22 | 225,907 |
| **Outcome-associated terms** | | |
| #24 | Mortality | 2,000,773 |
| #25 | Survival | 2,275,525 |
| #26 | All-cause mortality | 125,332 |
| #27 | #24 OR #25 OR #26 | 3,837,252 |
| **Combined term** | | |
| #31 | #8 AND #23 AND #27 | 136 |
| #32 | Filter: year of publications after 2000 | 131 |

**Supplementary Material 4.** Predefined worksheet for data collection.

| **Parameter** | **Unit** |
| --- | --- |
| Studyname | NA |
| Year | Year |
| Applied definition | NA |
| Intervention | CABG/OPCAB |
| Follow-up (mean) | Years |
| Follow-up (SD) | Years |
| Number of patients | n |
| Age (mean) | Years |
| Age (mean) | Years |
| Sex (male/female) | n |
| Pre-operative history | NA |
| Prior PCI | n |
| Prior CABG | n |
| LVEF (mean) | % |
| LVEF (SD) | % |
| Extent of coronary artery disease | NA |
| SYNTAX-score | n |
| EuroSCORE | % |
| Procedural characteristics | NA |
| Number of patients with PMI according to one definition | n |
| Number of events during follow-up in these patients (mortality) | n |
| Unadjusted hazard ratio for mortality during follow-up | HR |
| Unadjusted 95%CI | CI |
| Adjusted hazard ratio for mortality during follow-up | HR |
| Adjusted 95%CI | CI |

*CABG: coronary artery bypass grafting, CI: confidence intervals, EuroSCORE: European System for Cardiac Operative Risk Evaluation, HR: hazard ratio, LVEF: left ventricular ejection fraction, NA: not applicable, OPCAB: off-pump CABG, PCI: percutaneous coronary intervention, SD: standard deviation.*

**Supplementary Material 5.** PRISMA flowchart for study inclusion.

*CABG: coronary artery bypass grafting, PMI: periprocedural myocardial infarction, STEMI: ST-elevation myocardial infarction,*

**Supplementary Material 6.** Pre-operative patient characteristics (i).

| Study | Mean age (year) | Male (n) | BMI (kg/m2) | DM (n) | HT (n) | HL (n) | COPD (n) | RI (n) | PAD (n) | Dialysis (n) | Current smoker (n) | Stroke (n) | MI (n) | PCI (n) | CABG (n) |
| --- | --- | --- | --- | --- | --- | --- | --- | --- | --- | --- | --- | --- | --- | --- | --- |
| Belley-Cote, 2015 ^1^ | 68±7 | 3 843 | 26.7±4.4 | 2 228 | 3 604 | NR | NR | NR | 385 | 66 | NR | 456 | 1 641 | 463 | NR |
| Cho, 2017 ^2^ | 63.4± 8.9 | 2 336 | 24.8±3 | 1 391 | 2 023 | 638 | 63 | 139 | 139 | NR | 750 | 293 | 294 | 502 | 21 |
| Gregson, 2020 ^3^ | 66± 10 | 719 | NR | 256 | 220 | 207 | 77 | 137 | 83 | 3 | 187 | NR | NR | NR | NR |
| Hara, 2020 ^4^ | 64.9± 10 | 633 | 27.9±4.5 | 191 | 506 | 612 | 72 | 140 | 83 | NR | 175 | 119 | 263 | NR | NR |
| Hinton, 2022 ^5^ | NR | NR | NR | NR | NR | NR | NR | NR | NR | NR | NR | NR | NR | NR | NR |
| Jang, 2016 ^6^ | 62.4± 9.1 | 311 | NR | 219 | 235 | 125 | NR | 21 | NR | NR | 123 | 39 | 80 | 58 | NR |
| Litwinowicz, 2022 ^7^ | 66±9 | 3 610 | NR | 1 587 | 4 270 | 1 653 | 177 | 1 527 | NR | 26 | 719 | NR | 2 548 | NR | NR |
| Pölzl, 2022 ^8^ | 67± 9.6 | 2 316 | 27.3±1.5 | 843 | 2 529 | 2 098 | 302 | NR | NR | NR | NR | 53 | 931 | 430 | NR |
| Wang, 2013 ^9^ | 64.3± 9.8 | 450 | 29.1±5.3 | 147 | 389 | 512 | 101 | NR | 58 | 15 | 75 | 30 | 357 | 61 | 9 |
| Yau, 2008 ^10^ | 63.1± 10.2 | 2 385 | NR | 1 139 | 2 263 | 2 299 | 475 | 65 | 369 | NR | 690 | 165 | NR | 780 | NR |

*BMI: body mass index, CABG: coronary artery bypass grafting, COPD: chronic obstructive pulmonary disease, DM: diabetes mellitus, HT: hypertension, PAD: peripheral arterial disease, MI: myocardial infarction, NR: not reported, PCI: percutaneous coronary intervention, RI: renal insufficiency,*

**Supplementary Material 7.** Pre-operative patient characteristics (ii).

| Study | Mean LVEF (%) | 1 vessel disease (n) | II vessel disease (n) | III vessel disease (n) | CTO (n) | LM disease (n) | Syntax score | EuroSCORE |
| --- | --- | --- | --- | --- | --- | --- | --- | --- |
| Belley-Cote, 2015 | NR | 119 | 817 | 2711 | NR | 1001 | NR | 3.8 ± 2.3 |
| Cho, 2017 | 53±11.4 |  | 656 | 2527 | 316 | 984 | NR | NR |
| Gregson, 2020 | 58.5±7.7 | NR | NR | NR | NR | 657 | 25.5±10 | NR |
| Hara, 2020 | 58.1±13.2 | NR | NR | 590 | NA | 321 | 29.2±11.4 | NR |
| Hinton, 2022 | NR | NR | NR | NR | NR | NR | NR | NR |
| Jang, 2016 | 52.9±13.8 | NR | NR | NR | NR | NR | 28.9±10.6 | NR |
| Litwinowicz, 2022 | NR | NR | NR | NR | NR | 2467 | NR | 2.18±1.02 |
| Pölzl, 2022 | 60.7±23 | NR | NR | NR | NR | NR | NR | NR |
| Wang, 2013 | NR | NR | NR | 446 | NR | 240 | NR | 2.3±3.1 |
| Yau, 2008 | NR | 511 | 1095 | NR | NR | NR | NR | NR |

*CTO: chronic total occlusion, EuroSCORE: European System for Cardiac Operative Risk Evaluation, LM: left main, LVEF: left ventricular ejection fraction, NR: not reported.*

**Supplementary Material 8.** Procedural characteristics.

| Study | Elective surgery (n) | On-pump (n) | Off-pump (n) | Cross clamp time (min) | CPB time (min) | Blood cardioplegia (n) | Crystalloid cardioplegia (n) |
| --- | --- | --- | --- | --- | --- | --- | --- |
| Belley-Cote, 2015 | 2 910 | 2 377 | 2 375 | NR | NR | 1 987 | 175 |
| Cho, 2017 | NR | NR | NR | NR | NR | NR | NR |
| Gregson, 2020 | NR | NR | NR | NR | NR | NR | NR |
| Hara, 2020 | NR | 795 | NR | NR | NR | NR | NR |
| Hinton, 2022 | NR | NR | NR | NR | NR | NR | NR |
| Jang, 2016 | NR | 80 | 287 | NR | NR | NR | NR |
| Litwinowicz, 2022 | 3 774 | NR | NR | NR | NR | NR | NR |
| Pölzl, 2022 | NR | 2 828 | 1 | 63±23 | NR | NR | 2 829 |
| Wang, 2013 | 161 | 545 | 15 | 60±21 | 91±27 | 560 | 0 |
| Yau, 2008 | 1 461 | 2 377 | 637 | NR | 108.9± 35.7 | NR | NR |

*CPB: cardiopulmonary bypass, NR: not reported.*

**Supplementary Material 9.** Risk of bias assessment.

|  | Study arms | Selection | | | | Comparability | Outcome | | |
| --- | --- | --- | --- | --- | --- | --- | --- | --- | --- |
| *Questions** |  | 1 | 2 | 3 | 4 | 5 | 6 | 7 | 8 |
| *Studies* | | | | | | | | | |
| Belley-Cote, 2015**^1^** | 1 | Low | UC | Low | Low | Low | Low | Low | Low |
| Cho, 2017**^2^** | 2 | Low | Low | Low | Low | Low | Low | Low | Low |
| Gregson, 2020**^3^** | 2 | Low | Low | Low | Low | Low | Low | Low | Low |
| Hara, 2020**^4^** | 2 | Low | Low | Low | Low | Low | Low | Low | Low |
| Hinton, 2022**^5^** | 1 | Low | UC | Low | Low | Low | High | Low | Int |
| Jang, 2016**^6^** | 2 | Low | Low | Low | Low | Int | Low | Low | Low |
| Litwinowicz, 2022**^7^** | 2 | Low | Low | Low | Low | Low | Low | Low | Low |
| Pölzl, 2022**^8^** | 2 | Low | Low | Low | Low | Low | Low | Low | Low |
| Wang, 2013**^9^** | 3 | Low | Low | Low | Low | Low | Low | Low | Low |
| Yau, 2008**^10^** | 2 | Low | Low | Low | Low | Low | High | Low | Int |

*The items for the specific questions comprise: *Selection:* representativeness of the exposed cohort (1), selection of the non-exposed cohort (2), ascertainment of exposure (3), outcome of interest not present at the start of the study (4)*. Comparability:* Comparability of cohorts on the basis of design or analysis (5). *Outcome:* assessment of outcome (6), long enough follow-up (7), adequacy of follow-up (8).

*UC: unclear, Int.: intermediate.*

**Supplementary Material 10.** Frequentist meta-analysis of UDMI-3 (A) and UDMI-4 (B) separately.

**
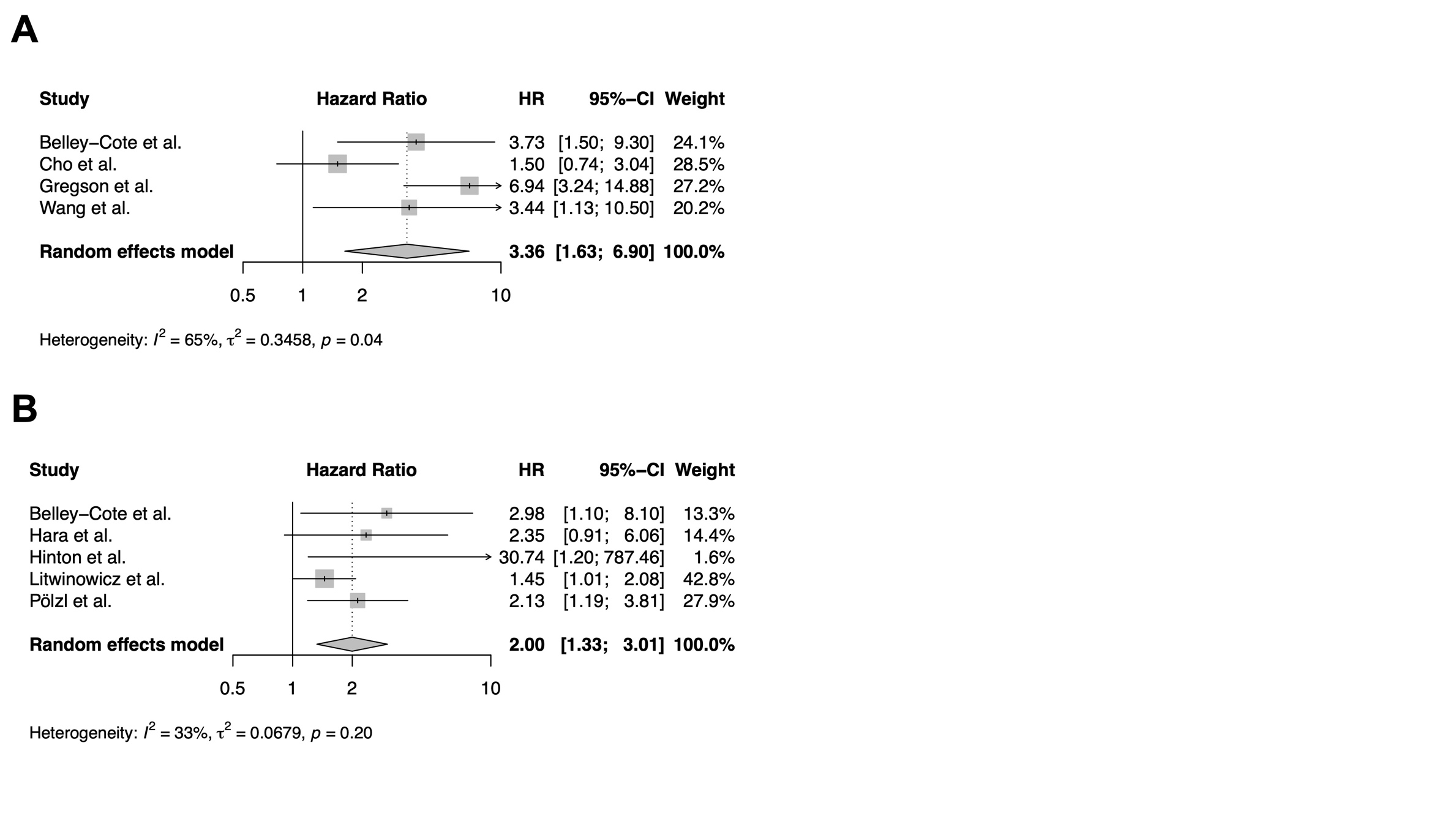
**

*CI: confidence intervals, HR: hazard ratio, UDMI: universal definition of myocardial infarction.*

**Supplementary Material 11.** Publication bias assessment.


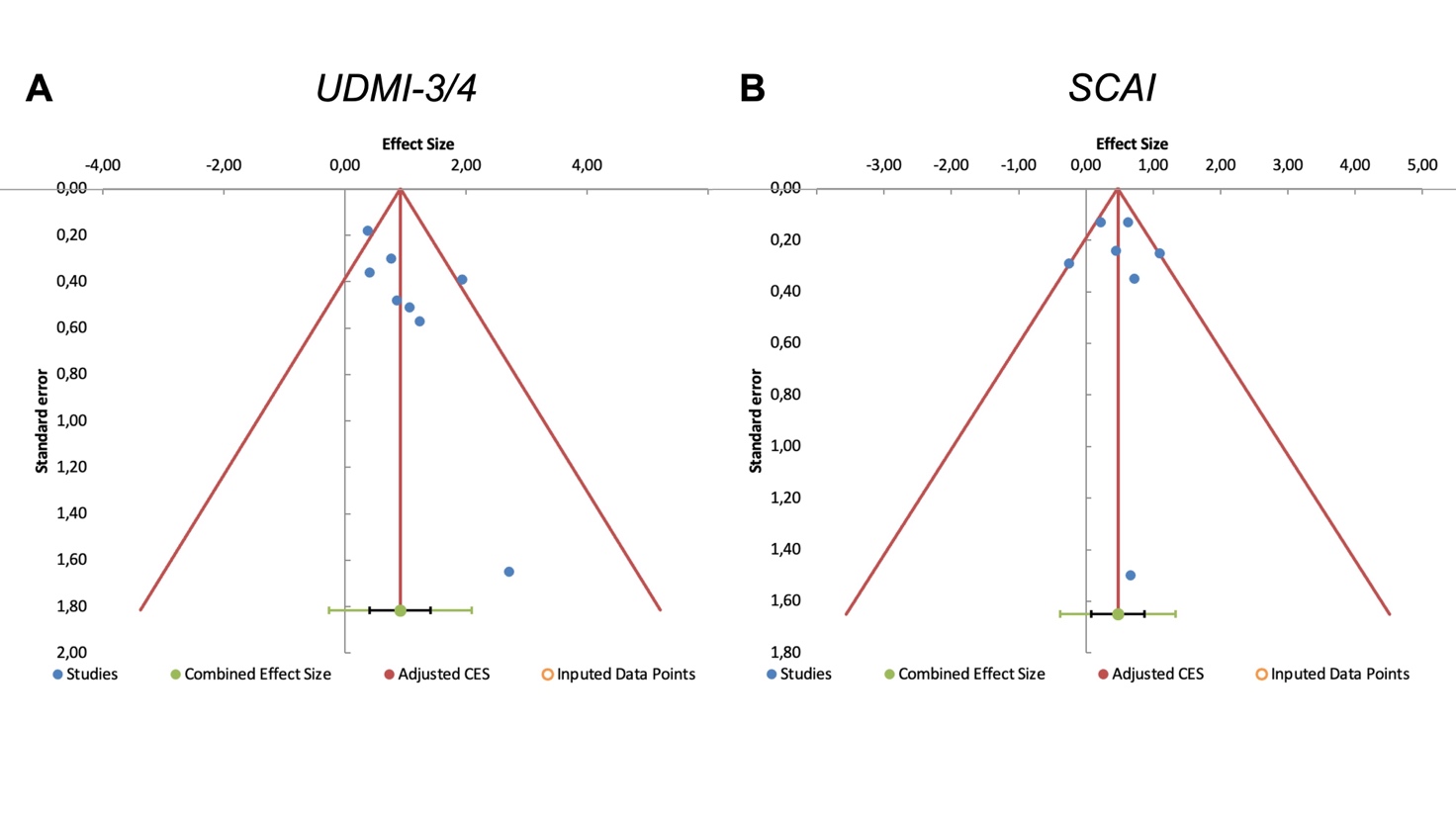


*CES: combined effect size, SCAI: society of cardiovascular angiography and interventions, UDMI: universal definition of myocardial infarction.*

REFERENCES FOR SUPPLEMENTARY MATERIAL

1. Belley-Cote EP, Lamy A, Devereaux PJ*, et al.* Definitions of post-coronary artery bypass grafting myocardial infarction: variations in incidence and prognostic significance. *Eur J Cardiothorac Surg* 2019;**57**:168–175. doi:

2. Cho MS, Ahn JM, Lee CH*, et al.* Differential Rates and Clinical Significance of Periprocedural Myocardial Infarction After Stenting or Bypass Surgery for Multivessel Coronary Disease According to Various Definitions. In. *JACC. Cardiovascular interventions*. 15 ed. United States; 2017, 1498-1507.

3. Gregson J, Stone GW, Ben-Yehuda O*, et al.* Implications of Alternative Definitions of Peri-Procedural Myocardial Infarction After Coronary Revascularization. 2020;**76**:1609‐1621. doi: 10.1016/j.jacc.2020.08.016

4. Hara H, Serruys PW, Takahashi K*, et al.* Impact of Peri-Procedural Myocardial Infarction on Outcomes After Revascularization. *Journal of the American College of Cardiology* 2020;**76**:1622-1639. doi: 10.1016/j.jacc.2020.08.009

5. Hinton J, Augustine M, Gabara L*, et al.* Incidence and 1-year outcome of periprocedural myocardial infarction following cardiac surgery: are the Universal Definition and Society for Cardiovascular Angiography and Intervention criteria fit for purpose? *Eur J Cardiothorac Surg* 2022;**62**. doi: 10.1093/ejcts/ezac019

6. Jang WJ, Yang JH, Choi SH*, et al.* Association of periprocedural myocardial infarction with long-term survival in patients treated with coronary revascularization therapy of chronic total occlusion. *Catheterization and Cardiovascular Interventions* 2016;**87**:1042-1049. doi: <https://dx.doi.org/10.1002/ccd.26286> PT - Article

7. Litwinowicz R, Mazur P, Śliwiński P*, et al.* Long-term survival following postoperative myocardial infraction after coronary artery bypass surgery. *Journal of thoracic disease* 2022;**14**:102-112. doi: 10.21037/jtd-21-1279

8. Pölzl L, Thielmann M, Cymorek S*, et al.* Impact of myocardial injury after coronary artery bypass grafting on long-term prognosis. *European heart journal* 2022;**43**:2407-2417. doi: 10.1093/eurheartj/ehac054

9. Wang TK, Stewart RA, Ramanathan T*, et al.* Diagnosis of MI after CABG with high-sensitivity troponin T and new ECG or echocardiogram changes: relationship with mortality and validation of the universal definition of MI. *European heart journal. Acute cardiovascular care* 2013;**2**:323-333. doi: 10.1177/2048872613496941

10. Yau JM, Alexander JH, Hafley G*, et al.* Impact of perioperative myocardial infarction on angiographic and clinical outcomes following coronary artery bypass grafting (from PRoject of Ex-vivo Vein graft ENgineering via Transfection [PREVENT] IV). 2008;**102**:546‐551. doi: 10.1016/j.amjcard.2008.04.069
